# Dehydrozaluzanin C inhibits colon cancer cell proliferation, apoptosis and cycle arrest through peroxisome proliferator-activated receptor γ (PPARγ) activation

**DOI:** 10.3389/fphar.2025.1623153

**Published:** 2025-09-11

**Authors:** Shan-Shan Li, Zhao-Ting Li, Xiao-Qing Zhu, Xu Li, Xi-Ke Xu, Xian-Peng Zu, Xian Li, Yun-Heng Shen

**Affiliations:** ^1^ Center of Clinical Pharmacy, First Affiliated Hospital of Kunming Medical University, Kunming, Yunnan, China; ^2^ Department of Phytochemistry, School of Pharmacy, Naval Medical University, Shanghai, China; ^3^ School of Pharmaceutical Sciences and Yunnan Key Laboratory of Pharmacology for Natural Products, Kunming Medical University, Kunming, Yunnan, China

**Keywords:** Dehydrozaluzanin C, colon cancer, apoptosis induction, cycle arrest, PPARγ activation

## Abstract

Dehydrozaluzanin C (DC) is a sesquiterpene lactone isolated from Asteraceae plant *Ainsliaea macrocephala*. To investigate the antitumor effects of DC and possible molecular mechanisms for treating cancer. The antitumor effect of DC was studied using HT-29 and HCT-116 human colon tumor cell lines and Balb/c nude mice models. The anti-proliferative, proapoptotic effects, and cycle arrest of DC were observed by cell viability, colony formation, apoptosis, and cycle assays. The changes of protein expression level were examined by Western blot analysis. The transcription activity of PPARγ was determined by Luciferase reporter assay. The role of PPARγ activation in the antitumor activity of DC was verified using PPARγ antagonist GW9662 and si-PPARγ HT-29 cells. DC treatment significantly decreased colon tumor cell viability, cell clone number, and increased apoptosis rate and arrested cell cycle at S phase. Furthermore, DC treatment significantly decreased Bcl-2, CDK2, and cyclin A2 protein levels while increasing the expression of cleaved caspase 3 and Bax in HT-29 and HCT-116 cells. Further investigations indicated that cell survival, induction of apoptosis, and cycle arrest by DC could be significantly reversed following treatment with the PPARγ antagonist GW9662 or in si-PPARγ cells. *In vivo*, DC treatment significantly decreased the weight and volume of xenograft tumor tissues in mice and apoptosis-related protein levels. The results suggest that DC effectively inhibits colon tumor cell proliferation, clone formation, apoptosis, and cell cycle arrest through PPARγ activation. These results support the potential of DC as an anti-tumor lead compound for further investigation.

## 1 Introduction

Colon cancer is one of the most commonly gastrointestinal cancers and the third leading cause of cancer deaths (Siegel et al., 2023). Despite significant improvements in treatment options, the overall survival rates of patients with colon cancer are still significantly lower than other cancer patients, with a 5-year median survival rate of less than 10% (Bray et al., 2018). As such, there is an urgent need for novel antitumor drugs to treat colon cancer, though significant barriers to many of these new therapies remain.

Inducing cell apoptosis and arresting cell cycle are the two major strategies for anti-cancer treatment. Many plant-derived natural chemotherapeutic drugs, for example, paclitaxel (Yang et al., 2020), a tetracyclic diterpenoid, could dose- and time-dependently induce a cytotoxic effect, selectively target microtubules, and cause cell cycle arrest at the G2/M phase. Vincristine (Dhyani et al., 2022), a vinca alkaloid isolated from *Catharanthus roseus*, could arrest the cell cycle through disruption of microtubule dynamics in cancer treatment. Etoposide (Hashimoto et al., 2012), a podophyllotoxin derivative, could lead to cell death by breaking single- and double-strands of cellular DNA and delaying a proportion of the cell cycle in the late S or early G2 phase.

PPARγ is one of the ligand-activated nuclear receptors in PPARs subfamily (Grommes et al., 2004). PPARγ play a vital role in glucose homeostasis (Anghel and Wahli, 2007), lipid metabolism (Semple, 2006), and inflammation (Moraes et al., 2006). Evidences have shown that PPARγ agonists could inhibit cell proliferation of several human tumor cell lines, including nasopharyngeal carcinoma (Yang et al., 2019), glioblastoma (Wang et al., 2021a), diffuse large B cell lymphoma (Zhang et al., 2020), bladder cancer (Lv et al., 2019), etc. PPARγ agonist include natural PPARγ ligands and synthetic thiazolidinediones (TZDs). However, the majority of TZDs exhibited PPARγ related adverse effects, including hepatotoxicity, congestive heart failure and fluid retention (Goltsman et al., 2016; Loke et al., 2011; Paul et al., 1998). The adverse effects of natural PPARγ ligands were rarely reported. Therefore, the discovery of new natural PPARγ ligands with selective therapeutic activity and fewer side effects will be beneficial for cancer treatment (Atanasov et al., 2021).

**FIGURE 1:**
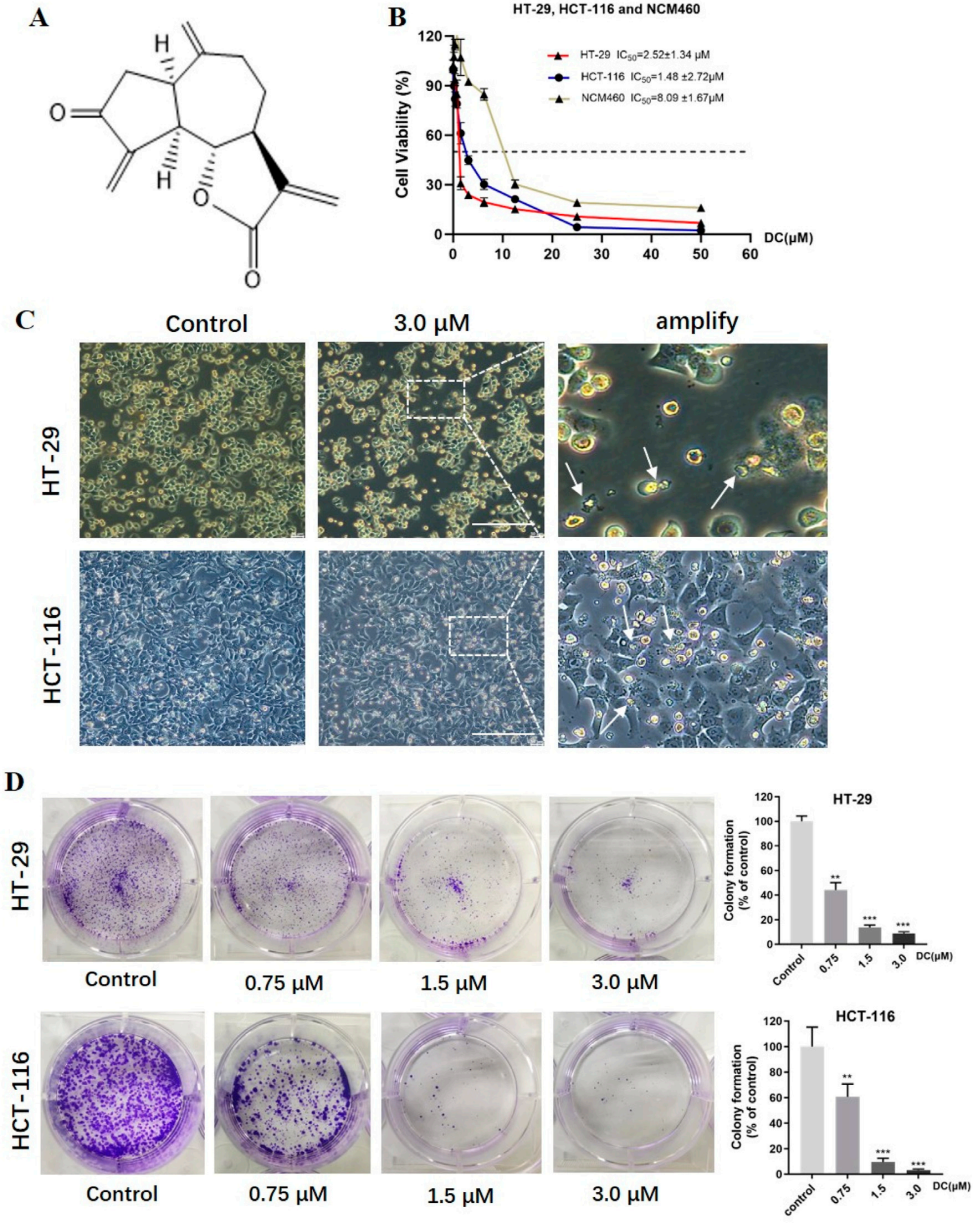
DC suppressed cell proliferation and colony formation in colon cancer cells. **(A)** The chemical structure of DC. **(B)** HT-29, HCT-116 and NCM460 cells were treated at the indicated concentration of DC for 24 h, and cell viability was measured by CCK-8 assay (n = 6). **(C)** Typical morphological change by DC treatment in HT-29 and HCT-116 cells observed under an inverted light Microscope (Bar = 200 μm). The white arrows indicated that the cells are solidified or fragmented into several granules. **(D)** The cells were treated by the indicated concentration of DC and stained with 0.5% crystal violet, and photographed with a digital camera. Quantitative analysis of colony formation of HT-29 and HCT-116 cells, respectively (n = 3). Significance was determined by the one-way ANOVA (^***^ *P* < 0.001, ^**^ *P* < 0.01, ^*^ *P* < 0.05 vs. control).

Natural sesquiterpenoids have been considered as a class of potential compounds for the development of novel anticancer agents. Dehydrozaluzanin C (DC, the chemical structure as shown in Figure 1A) is a guaiane sesquiterpene lactone isolated from Asteraceae plant *Ainsliaea macrocephala* (Wu et al., 2011). Previous studies have shown that DC has a variety of pharmacological effects, including antifungal (Wedge et al., 2000), anti-inflammatory (Lajter et al., 2015), and anti-proliferative activities (Macıas et al., 2000). The studies in our group have reported a series of dimeric and trimeric derivatives of DC from *Ainsliaea* species, including ainsliadimer A (Wang et al., 2008) and ainsliatrimer B (Wu et al., 2008), and Chao Li et al. found that ainsliatrimer A could remarkably suppress the proliferation of Hela cells by PPARγ activation (Li et al., 2014). However, the cytotoxic activity of DC on tumor cells and the underlying antitumor mechanism remain rarely studied.

In this study, we evaluated the cytotoxic activity of DC in two tumor cell lines and examined its effects on cell proliferation, colony formation, apoptosis, and cycle arrest. Additionally, we explored the possible molecular mechanisms underlying its action in colon cancer cells.

## 2 Materials and methods

### 2.1 Reagents, chemicals, and cell culture

The DC was synthesized and identified in our lab (purity > 98%). DC was dissolved in DMSO (Sigma-Aldrich, United States) to prepare a solution of 10 mM and stored at −20 °C. Rog (rosiglitazone) was purchased from Dalian Meiluo Biotechnology Co., Ltd. GW9662 were purchased from Selleck Chemicals (S2915). NCM460, HT-29 and HCT-116 cells were kindly provided by Stem Cell Bank, Chinese Academy of Sciences, and were cultured in McCoy’s 5A medium (12330031, Gbico) supplemented with 10% fetal bovine serum (16140071, Gbico) at 37 °C and 5% CO_2_.

### 2.2 Cell viability assay

Cell viability was measured using the CCK-8 assay. Cells were seeded in 96-well plates at a density of 1 × 10^4^ cells/well for 24 h. After treatment with the indicated concentration of DC for 24 h, then 10 μL of CCK-8 solution was added, and the plate was incubated for 1 h at 37 °C. Absorbance was measured at 450 nm using a BioTek Spectrum spectrophotometer (Thermo Scientific, United States). The IC_50_ value was calculated using GraphPad Prism7 software.

### 2.3 Colony formation assay

HT-29 and HCT-116 cells were planted on 6-well plates at a density of 1,000 cells/well for 24 h. The DC at different concentrations (0, 0.75, 1.5, and 3.0 μM) were added and cultured for 24 h. The culture medium was refreshed every 2 days to maintain growth for 10 days. The colonies were then washed with PBS, fixed with 4% paraformaldehyde, and stained with 0.1% crystal violet for 20 min at room temperature, followed by washing cells 3 times with PBS. Finally, the number of cell clones was counted using ImageJ software.

### 2.4 Cell apoptosis assay

Apoptosis was quantified by using an apoptosis detection kit (BD Biosciences, Tokyo, Japan) according to the manufacturer’s instructions. Cells (2 × 10^5^/mL) were seeded into 6-well plates and were treated with DC (0, 0.75, 1.5, and 3.0 μM) for 24 h. After incubation, cells were washed twice with PBS before harvesting and re-suspended in binding buffer. Annexin V-FITC/PI staining was added to the cell suspension and incubated for 15 min in the dark at 37 °C. Cells were analyzed using an ACEN flow cytometer. Early and late apoptosis were summed to calculate the apoptotic rate.

### 2.5 Cell cycle assay

Cell cycle was detected using a cell cycle analysis kit (BD Biosciences, United States) according to the manufacturer’s instructions. Cells (2 × 10^5^/mL) were seeded into 6-well plates and cultured for 24 h, and then were treated with the indicated DC concentration for 24 h. Then cells were washed twice with PBS, and then fixed in 70% ethanol for overnight at 4 °C. After that, cells were then stained with PI and analyzed cell cycle phase using an ACEN flow cytometer (ACEN, NovoCyte).

### 2.6 Luciferase activity assay

HT-29 cells (1 × 10^4^ cells/well) were co-transfected with 0.2 μg PPRE-TK-Luc reporter plasmid, and phRL-CMV Renilla luciferase in 24-well plates using Lipofectamine 2000 (Invitrogen, 11668019) for 24 h. Co-transfected cells were then treated with Rog or DC with or without GW9662 for 24 h. Cells were harvested, and luciferase activity was measured using the dual-Luciferase reporter assay system (Promega, E1980) according to the manufacturer’s instructions. Firefly luciferase values were divided by Renilla luciferase values to control for transfection efficiency.

### 2.7 Molecular docking

Molecular docking was performed using AutoDock Vina 1.1.2 software (The Scripps Research Institute, La Jolla, CA, United States). Default settings and the Vina scoring function were employed. The crystal structure of PPARγ was obtained from the Protein Data Bank (PDB ID: 2PRG). Ligands and water molecules were removed from the crystal structures of the protein, and hydrogen atoms were added. Analysis and visual exploration of the ligand-protein interactions of the docking poses was performed using Discovery Studio 2020 software (Dassault Systems BIOVIA, San Diego, CA, United States, 2020).

### 2.8 Cell transfection using RNA interference

Lipofectamine™ RNAiMAX (Invitrogen, 13778150) was purchased from Thermo-Fisher Scientific. The anti-PPARγ siRNA (si-PPARγ: 5-CCAAGUUUGAGUUUGCUGUdTd-3) (Li et al., 2014) and negative control siRNA (si-NC: 5-CCUAGUAUGACUAAGCUGUdTd-3) were designed and synthesized by GenePharma (Shanghai, China). HT-29 cells were transiently transfected for 48 h according to the manufacturer′s instructions (Lifetechnologies). After 48 h of transfection, the cells were examined and used for the subsequent assay.

### 2.9 HT-29 cell xenograft tumor in mice

Four-week-old male Balb/c nude mice were obtained from BiKai Biotechnology Co., Ltd. (Shanghai, China) and were housed and maintained under specific-pathogen-free (SPF) conditions in facilities approved by the Animal Ethics Committee of the Naval Medical University. All animal experiments were conducted in accordance with the Guide for the Care and Use of Laboratory Animals of the National Institutes of Health and approved by the Animal Ethics Committee of the Naval Medical University, China (Approval No. SMMU-2022-13). Each animal was injected subcutaneously with HT-29 cells (2 ×10^6^ cells/100 μL) into the right flanks of the nude mice. When the tumors reached a size of 100 mm^3^, the mice were randomly divided into 3 groups (6 mice/group) and treated with saline containing 0.9% sodium chloride (vehicle) or DC at doses of 1.5 mg/kg and 3.0 mg/kg by intraperitoneal injection once a day for 14 consecutive days. Tumor size was measured every other day using calipers, and tumor volume was calculated according the formula: tumor volume (mm^3^) = (tumor length) × (tumor width)^2^/2. Mice were sacrificed after 14 days of treatment. All procedures were conducted in accordance with the accepted guidelines for the use and care of laboratory animals. The tumors were harvested, photographed, weighed, and then stored at −80 °C for subsequent experiments.

### 2.10 HE staining

Heart, liver, spleen, lung, kidney and tumor tissues from nude mice were fixed in 4% paraformaldehyde and embedded in paraffin. The paraffin-embedded tumor tissue sections (5 μm) were deparaffinized and rehydrated before staining with eosin and hematoxylin. The images were captured by using a light microscope (Leica, DMi8).

### 2.11 Tunnel and ki67 staining

Deparaffinized and rehydrated tissue sections were permeabilized with 0.5% Triton X-100 and incubated with normal goat serum for 1 h at 37 °C. All washes between each step were performed with TBS. Detection of apoptotic cells was performed using the tunnel assay kit (Servicebio, G1501) and the proliferation marker was examined with the Ki67 antibody (Servicebio, GB121141) in tumor tissues according to the manufacturer’s instructions, and nuclei were counterstained with 5 μg/mL DAPI (Servicebio, G1012) for 5 min at 37 °C. Images were captured using a light microscope (Leica, DMi8).

### 2.12 Western blot

Drug-treated cells and tumor tissues from xenograft mice were harvested and washed twice with ice-cold PBS. Cells and tumor tissues were lysed with RIPA buffer (Beyotime, P0013C) containing 1x protease inhibitors (Roche) and centrifuged (12,000 g for 15 min) at 4 °C. The supernatant was collected and the protein concentration was determined by the BCA assay (Beyotime, P0011). Equivalent amounts of protein (15–30 μg) were loaded and separated on 10% SDS-PAGE gels. After electrophoresis, protein bands were transferred to PVDF membranes (Bio-Rad) and blocked with 5% non-fat milk for 1 h at 37-°C. Bands were incubated overnight with an appropriate primary antibody at 4-°C. The primary antibodies were as follows: cleaved caspase 3 (CST, 9,661), Bax (Abcam, ab53154), Bcl-2 (Abcam, ab196495), CDK2 (CST, 18,048), Cyclin A2 (CST, 67,955), and β-actin (CST, 3,700). The next day, the membrane was washed three times with TBST for 5 min each and incubated again with the secondary antibody for 1 h at room temperature. β-Actin was also loaded as a control. Images were acquired from LI-COR (Lincoln, NE, United States). Band intensity was quantified using ImageJ software.

### 2.13 Statistical analysis

Data were calculated as the mean ± SD of at least three independent experiments. Statistical analysis was performed using the GraphPad Prism 7.0 software (San Diego, CA, United States). Student’s t-test was used to compare differences between two groups. Differences between multiple groups were analyzed by one-way ANOVA with Tukey’s *post hoc* test. Differences were considered to be significant when *p* < 0.05.

## 3 Results

### 3.1 Effects of DC on cytotoxicity and colony formation of HT-29 and HCT-116 cells

To investigate the antitumor effect of DC on human tumor cell lines. A cytotoxicity assay was conducted using CCK-8. In previous experiments, our results have shown that DC has the strongest cytotoxicity in HT-29 cells, followed by MCF-7 and DU145 tumor cells (Supplementary Figure S1). So, human colon cell lines were chosen as experimental cells. As shown in Figure 1B, DC inhibited the proliferation of HT-29 and HCT-116 colorectal tumor cells, as well as NCM460 normal colon mucosal cells, in a concentration-dependent manner, with the IC_50_ values of 2.52 ± 1.34 μM, 1.48 ± 2.72 μM and 8.09 ± 1.67 μM, respectively, after incubation for 24 h. This result demonstrated that, compared to the colorectal tumor cells, DC exhibited lower cytotoxicity in the normal NCM-460 cells, with 3.21- to 5.47-fold higher viability in normal cells than in tumor cells, as shown in the dose-response curves. After DC treatment at 3.0 μM, the significant morphological changes were also observed, including the gradually shrinking cell bodies, larger gap, and small vesicles filled in the cytoplasm (Figure 1C). Additionally, to examine the ability of the cell lines to form a colony, HT-29 and HCT-116 cells were seeded and incubated with different concentrations of DC for 10 days. The results showed that colony formation of HT-29 and HCT-116 cells was suppressed by DC treatment in a dose-dependent manner. The rates of colony formation after DC (0, 0.75, 1.5 and 3.0 μM) treatment were 100.00% ± 3.55%, 44.16% ± 4.97%, 13.75% ± 1.54% and 8.95% ± 1.09% in HT-29 cells and were 100.00% ± 12.45%, 60.80% ± 8.11%, 9.51% ± 2.45% and 3.13% ± 0.85% in HCT-116 cells, respectively (Figure 1D). Collectively, DC exerted a potent inhibitory effect on the proliferation of human colon cell lines.

### 3.2 Effects of DC on apoptotic rate and the apoptosis-related protein expression in HT-29 and HCT-116 cells

**FIGURE 2:**
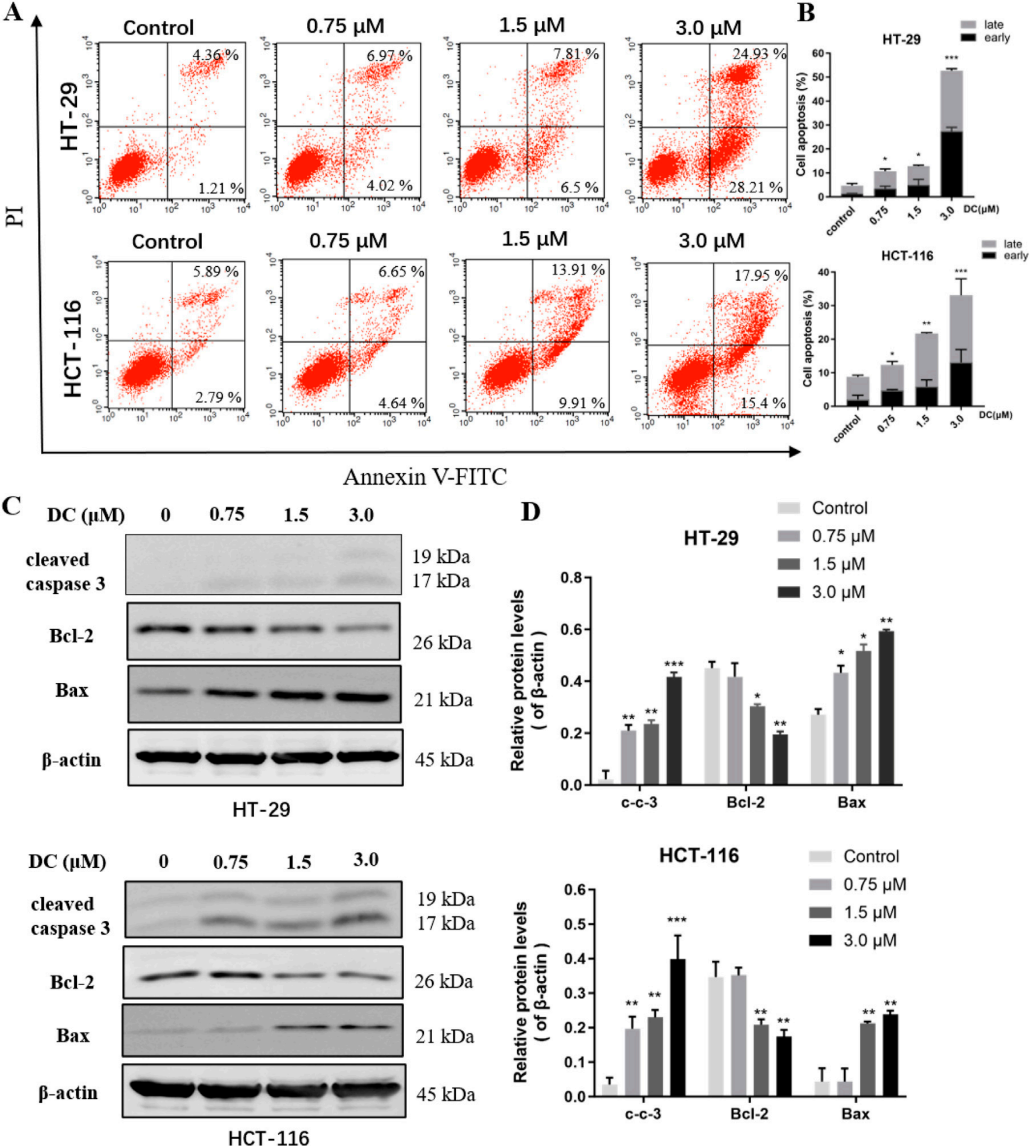
DC induced cell apoptosis and related-protein expression in colon cancer cells. **(A,B)** HT-29 and HCT-116 cells were treated with the indicated concentrations of DC for 24 h. The captured cells were fixed and stained by Annexin-V/FITC and PI to analyze the cell apoptotic rates using a flow cytometer. **(C,D)** The expression of cleaved caspase 3, Bcl-2, and Bax in DC-treated cells for 24 h by Western blotting. Data are expressed as the mean ± S.D. of three independent experiments. Significance was determined by the one-way ANOVA (^***^ *P* < 0.001, ^**^ *P* < 0.01, ^*^ *P* < 0.05 vs. control).

Induction of apoptosis in cancer cells is one of the major strategies for the development of antitumor drugs. To determine whether growth inhibition of DC was associated with cell apoptosis, HT-29 and HCT-116 cells were treated with DC (0, 0.75, 1.5, and 3.0 μM) for 24 h and then apoptosis rates were analyzed by flow cytometry. The results showed that DC obviously induced apoptosis rates to 52.67% ± 0.80% in HT-29 and 33.09% ± 1.09% in HCT-116 after 3.0 μM DC treatment, respectively (Figures 2A,B). The apoptotic induce effects were also verified by Western blotting, in which the protein expression levels of cleaved caspase 3 and Bax were elevated, while the protein level of Bcl-2 was significantly decreased following the indicated DC treatment (Figures 2C,D). These results revealed that DC could markedly induce colon tumor cell apoptosis.

### 3.3 Effects of DC on the cell cycle and cycle-associated protein expression in HT-29 and HCT-116 cells

**FIGURE 3:**
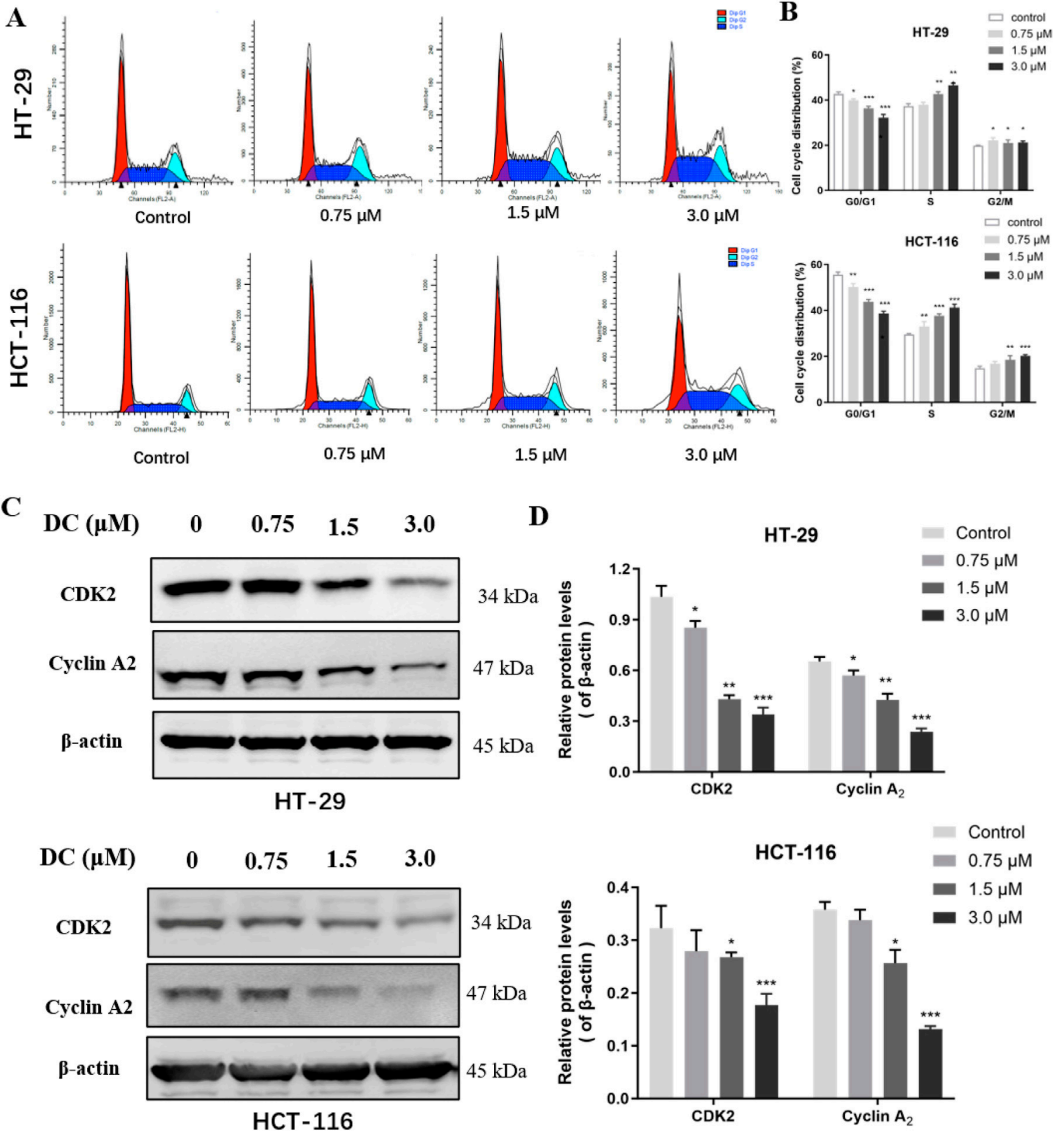
DC treatment arrested cell cycle at the S phase and regulated the expression of related proteins in colon cancer cells. **(A)** HT-29 and HCT-116 cells were treated with DC for 24 h. The cells were fixed and stained by PI to analyze the cell cycle distribution using a flow cytometer. **(B)** Quantification of the cell cycle distribution of HT-29 and HCT-116 cells. **(C,D)** The expression of CDK2 and Cyclin A2 followed treatment with DC for 24 h by Western blotting. Data are expressed as the mean ± S.D. of three independent experiments. Significance was determined by the one-way ANOVA (^***^ *P* < 0.001, ^**^ *P* < 0.01, ^*^ *P* < 0.05 vs. control).

Cell cycle dysregulation is crucial for the aberrant proliferation of tumor cells. To confirm the relationship between the growth inhibition of DC and cell cycle arrest, HT-29 and HCT-116 cells were treated with DC (0, 0.75, 1.5, and 3.0 μM) for 24 h and the cell percentage of each cycle phase was analyzed by flow cytometry. Our results showed that DC significantly induced cell cycle arrest at S phase, and the percentage in S phase was obviously higher in the 3.0 μM DC-treated group compared with the control group in HT-29 (46.53% ± 0.78 versus 37.39% ± 0.83) and HCT-116 (41.21% ± 1.25 versus 29.55% ± 0.29) cells, respectively. And compared with those of the control group, the percentage in G_0_/G_1_ phase was reduced in the HT-29 and HCT-116 cells, and the G_2_/M phase was elevated in the HT-29 and HCT-116 cells. The results suggested that DC induced cell cycle arrest at the S phase in a dose-dependent manner (Figures 3A,B). Furthermore, the protein expressions of the crucial mitotic signaling pathway in S phase arrest (CDK2 and cyclin A2), which are involved in the progression from S to G2/M phase were measured. The results demonstrated that the expressions of CDK2 and cyclinA2 were significantly reduced by DC at 3.0 μM in HT-29 and HCT-116 cells, further confirming that DC could arrest the cell cycle at S phase in colon cells (Figures 3C,D).

### 3.4 DC activated PPARγ transcription and molecular docking study

**FIGURE 4:**
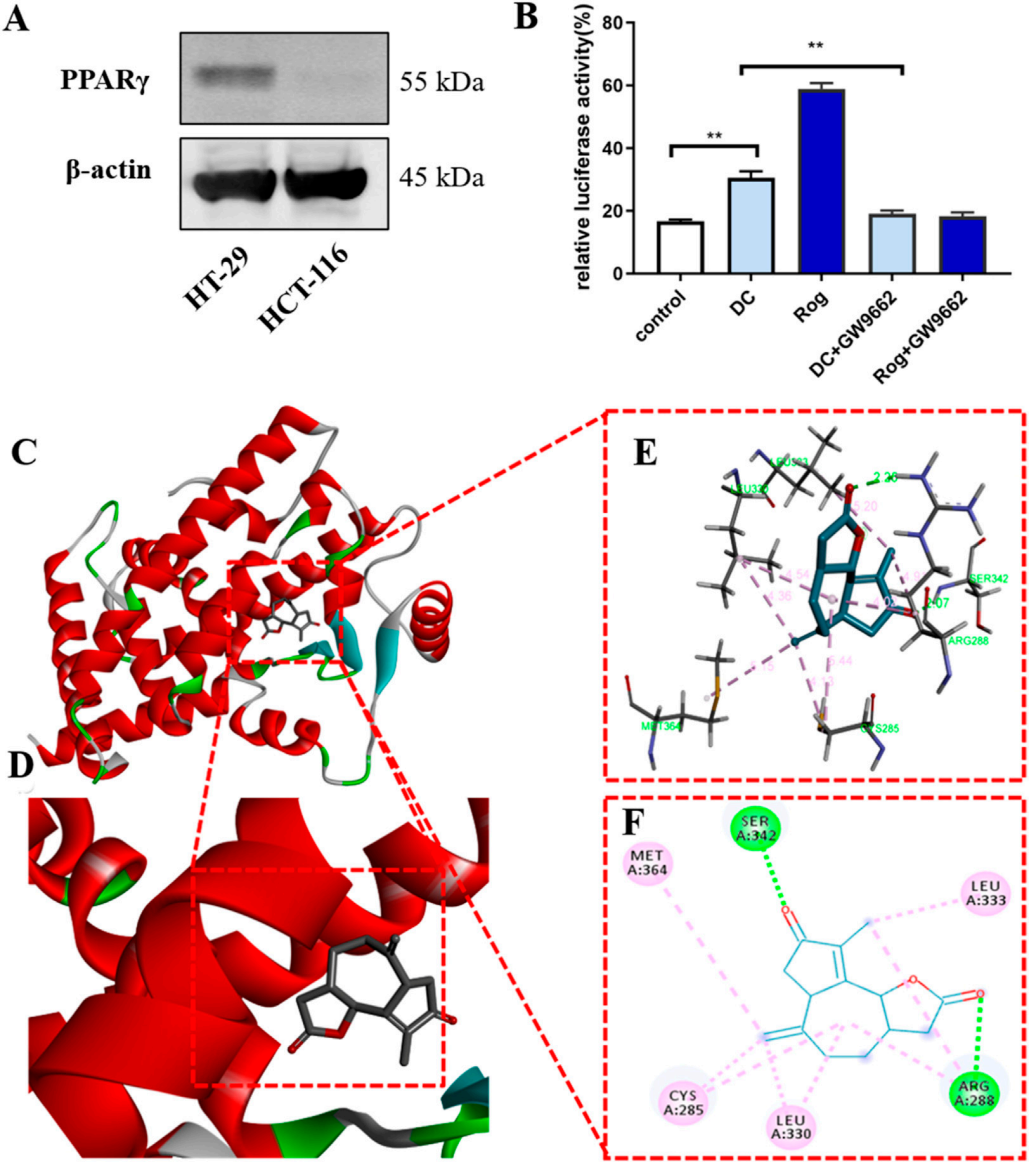
**(A)** The protein expression of PPARγ in HT-29 and HCT-116 cells. **(B)** DC increases the transcriptional activity of PPARγ (^***^ *P* < 0.001, ^**^ *P* < 0.01). **(C–F)** The 3D putative binding modes of DC with the ligand-binding domain (LBD) of PPARγ. **(C,D)** DC interacts with the key amino acid residues in PPARγ binding pocket (−7.9 kcal/mol). **(E,F)** DC interact with the Cys^285^, Leu^330^, Arg^288^, Leu^333^, Ser^342^, and Met^364^ in PPARγ binding pocket.

To test whether the antitumor effect of DC could be mediated by PPARγ transcription, a cell highly expressing endogenous PPARγ was chosen from the HT-29 and HCT-116 cells. As shown in Figure 4A, HT-29 expressed a higher PPARγ protein than HCT-116 cells, thus being chosen for subsequent experiments. Moreover, the luciferase reporter was constructed to examine PPARγ transcriptional activity following DC or rosiglitazone (Rog, a known PPARγ agonist, positive control) treatment. The results indicated that treatment with DC after 24 h, the transcriptional level of PPARγ in HT-29 cells was significantly enhanced by 1.81-fold (P < 0.01) than the control cells. To confirm the above observation, a selective PPARγ antagonist GW9962 was co-cultured with DC in HT-29, the result exhibited that the DC activated transcriptional activity of PPARγ was markedly attenuated, suggesting that the antitumor mechanism of DC might be associated with PPARγ activation (Figure 4B).

Docking studies were employed to explore the possibility of DC binding to PPARγ and the potential binding mode between PPARγ (PDB ID: 2PRG) and DC using AutoDock Vina 1.1.2 software. The results indicated that DC can tightly occupy the ATP binding site of PPARγ as shown in Figures 4C–F, with the score of −7.9 kcal/mol. The ester carbonyl group and ketone carbonyls of the five-membered ring of DC can strongly interact with the residues Arg^288^ and Ser^342^ in the hinge domain of PPARγ via two hydrogen bonds, respectively. Additionally, the seven-membered ring, double bond and methyl of DC can form hydrophobic interaction with Cys^285^, Leu^330^, Leu^333^, and Met^364^ residues.

### 3.5 PPARγ antagonist GW9662 abolished DC-mediated anti-proliferation, cell apoptosis induction and cell cycle arrest in HT-29 cells

**FIGURE 5:**
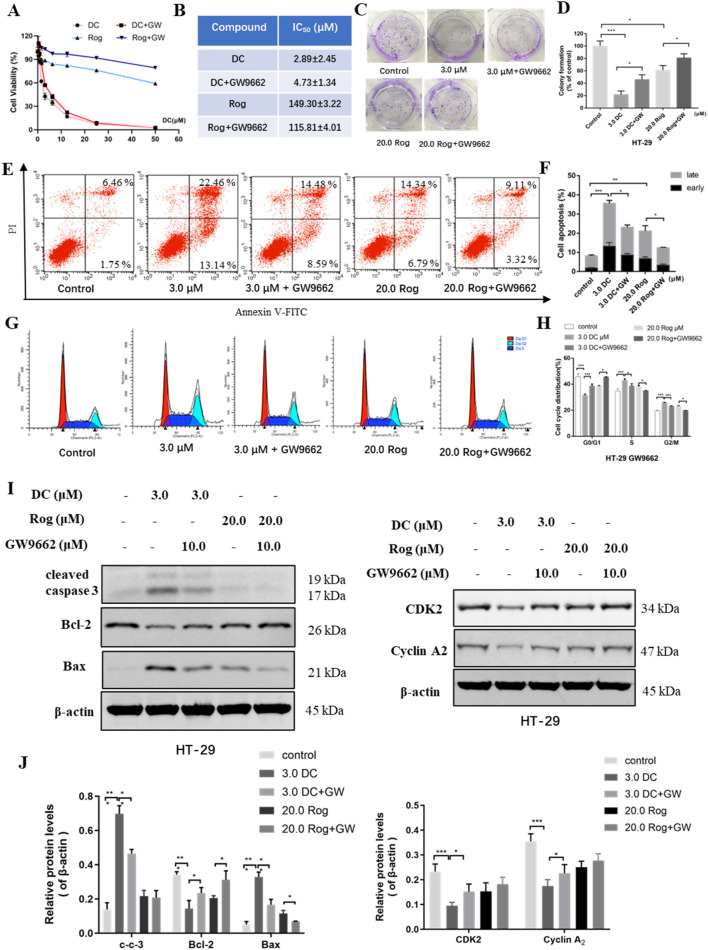
The effect of PPARγ antagonist (GW9662) for DC-mediated antiproliferation, cell colony formation, apoptosis induction and cell cycle arrest in HT-29 cells. HT-29 cells were co-treated with GW9662 and DC for 24 h **(A,B)** The anti-growth effect of DC was examined by CCK-8 and IC_50_ value was calculated in HT-29 cells. **(C,D)** Representative images of colony formation and quantitative analysis of colony-forming ability in HT-29 cells co-treated with GW9662 and DC. **(E,F)** The apoptosis of HT-29 cells co-treated with GW9662 and DC was determined by using annexin V/PI staining. Quantitative analysis of apoptotic effects in HT-29 cells (n = 3). **(G,H)** HT-29 cells were stained with PI for the detection of cell cycle distribution. The percentage of cells in each cycling phase is represented by the quantitative analysis (n = 3). **(I,J)** The protein levels of cleaved caspase-3, Bax, Bcl-2, CDK2 and Cyclin A2 were determined by Western blotting in HT-29 cells. Data are expressed as the mean ± S.D. of three independent experiments. Significance was determined by the one-way ANOVA (^***^ *P* < 0.001, ^**^ *P* < 0.01, ^*^ *P* < 0.05 vs. control).

To verify the role of PPARγ in DC-mediated proliferation inhibition, HT-29 cells were co-treated with DC and GW9662 (10.0 μM, a PPARγ antagonist). As shown in Figures 5A,B, compared to DC-treated group, the inhibitory effect of co-treatment was markedly reduced, suggesting that GW9662 blocked the growth inhibition of DC in HT-29 cells. Similarly, the co-treatment of DC and GW9662 could increase the number of HT-29 cell clones, indicating that PPARγ antagonist could abrogate the inhibition of DC on HT-29 cell colony formation (Figures 5C,D).

Previous experiments have revealed that PPARγ activation could induce cell apoptosis. To explore whether PPARγ activation was involved in the DC-induced apoptosis of HT-29 cells, the apoptosis rate was tested following co-treatment. Compared to the DC-treated group, the apoptotic rates of HT-29 cells co-treated with DC and GW9662 were sharply decreased from 35.59% ± 2.83% to 23.06% ± 1.60%, indicating that GW9662 treatment attenuated DC-induced apoptosis of HT-29 cells (Figures 5E,F). In the cell cycle assays, the cell proportion at S phase was obviously reduced from 42.89% ± 1.11% (3.0 μM DC) to 38.67% ± 1.43% (3.0 μM DC + GW9662) and that at G2/M phase from 25.75% ± 0.37% (3.0 μM DC) to 22.85% ± 0.55% (3.0 μM DC + GW9662), while the proportion at G0/G1 was markedly increased from 31.36% ± 0.90% to 38.48% ± 1.38%, suggesting that GW9662 treatment abrogated DC-induced S phase arrest of HT-29 cells (Figures 5G,H). Consistent with the results from the flow cytometry assay, Western blot showed that the protein levels of cleaved caspase-3 and Bax were decreased and those of Bcl-2, CDK2 and cyclin A2 were increased in HT-29 cells after co-treatment of DC and GW9662 (Figures 5I,J), suggesting that GW9662 treatment decreased DC-induced apoptosis and cell cycle-related protein expression in HT-29 cells.

### 3.6 The affection of PPARγ knockdown on DC-mediated anti-proliferation, cell apoptosis induction and cell cycle arrest in HT-29 cells

**FIGURE 6:**
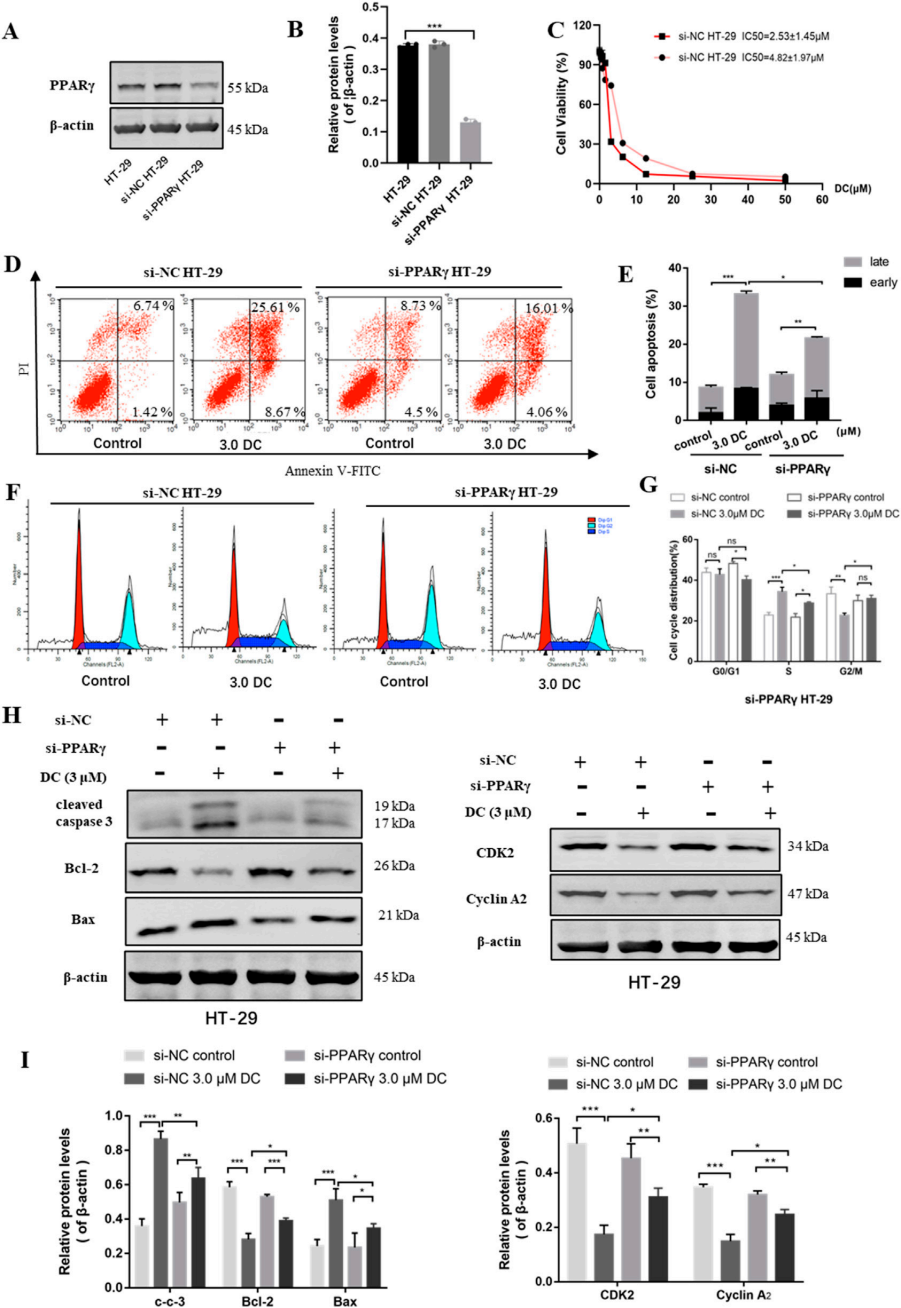
The effect of PPARγ knockdown for DC-mediated anti-proliferation, cell colony formation, apoptosis induction and cell cycle arrest in HT-29 cells. The si-NC or si-PPARγ HT-29 cells were treated with DC for 24 h **(A,B)** The relative expression of PPARγ in si-NC or si-PPARγ HT-29 cells were examined using WB. **(C)** The antiproliferation effect of DC was examined in PPARγ knockdown HT-29 cells. **(D,E)** The apoptosis induction of DC was examined by flow cytometry and cell apoptosis rate was calculated. **(F,G)** The cell cycle distribution was detected by flow cytometry, and the percentage of cells in each cycling phase was represented in the knockdown cells. **(H,I)** The protein level of cleaved caspase-3, Bax, Bcl-2, CDK2 and Cyclin A2 were determined by Western blotting in PPARγ knockdown HT-29 cells. Data are expressed as the mean ± S.D. of three independent experiments. Significance was determined by the one-way ANOVA (^***^ *P* < 0.001, ^**^ *P* < 0.01, ^*^ *P* < 0.05 vs. control).

To further confirm the role of PPARγ activation in the antitumor activity of DC, a si-PPARγ HT-29 cell line was constructed using RNA interference technology. The knockdown efficiency of PPARγ in HT-29 cells was examined using WB in si-NC HT-29 and PPARγ siRNA HT-29 cells. The results indicated that the endogenous PPARγ protein expression in si-PPARγ HT-29 cells were efficiently weakened compared to the control cells (Figures 6A,B). The si-NC HT-29 and si-PPARγ HT-29 cells were examined for cell viability, cell apoptosis, and cell cycle arrest by DC treatment.

Cell viability assay showed that the anti-proliferation effect of DC was markedly reduced in si-PPARγ HT-29 cells (Figure 6C), suggesting that the knockdown of PPARγ protein could reverse DC-mediated growth inhibition in HT-29 cells.

The apoptosis induction of DC against PPARγ siRNA HT-29 cells was examined by flow cytometry. As shown in Figures 6D,E, after DC treatment at the same concentrations, the apoptosis rate of si-PPARγ HT-29 cells (21.68% ± 1.69%) were markedly lower than that of si-NC HT-29 cells (33.24% ± 0.83%). In the cell cycle assay, compared to si-NC HT-29 cells, the proportion of PPARγ siRNA HT-29 cells at S phase were significantly reduced from 34.38% ± 1.78% to 28.8% ± 0.45%, while the proportion at G2/M was markedly increased from 22.73% ± 0.86% to 30.96% ± 1.37% after 3.0 μM DC treatment. The proportion of G0/G1 phase showed no obvious change (Figures 6F,G). This result suggested that the knockdown of PPARγ was able to reverse DC-mediated cell cycle arrest in HT-29 cells. Western blot showed that the protein levels of cleaved caspase-3 and Bax were decreased and the Bcl-2, CDK2, and cyclin A2 were increased in si-PPARγ HT-29 when compared with those of si-NC HT-29 group (Figures 6H,I), revealing that the knockdown of PPARγ could reverse DC-mediated apoptosis and cyclin-related protein expressions in HT-29 cells.

### 3.7 DC suppressed tumor growth in a xenografted nude mouse model

**FIGURE 7:**
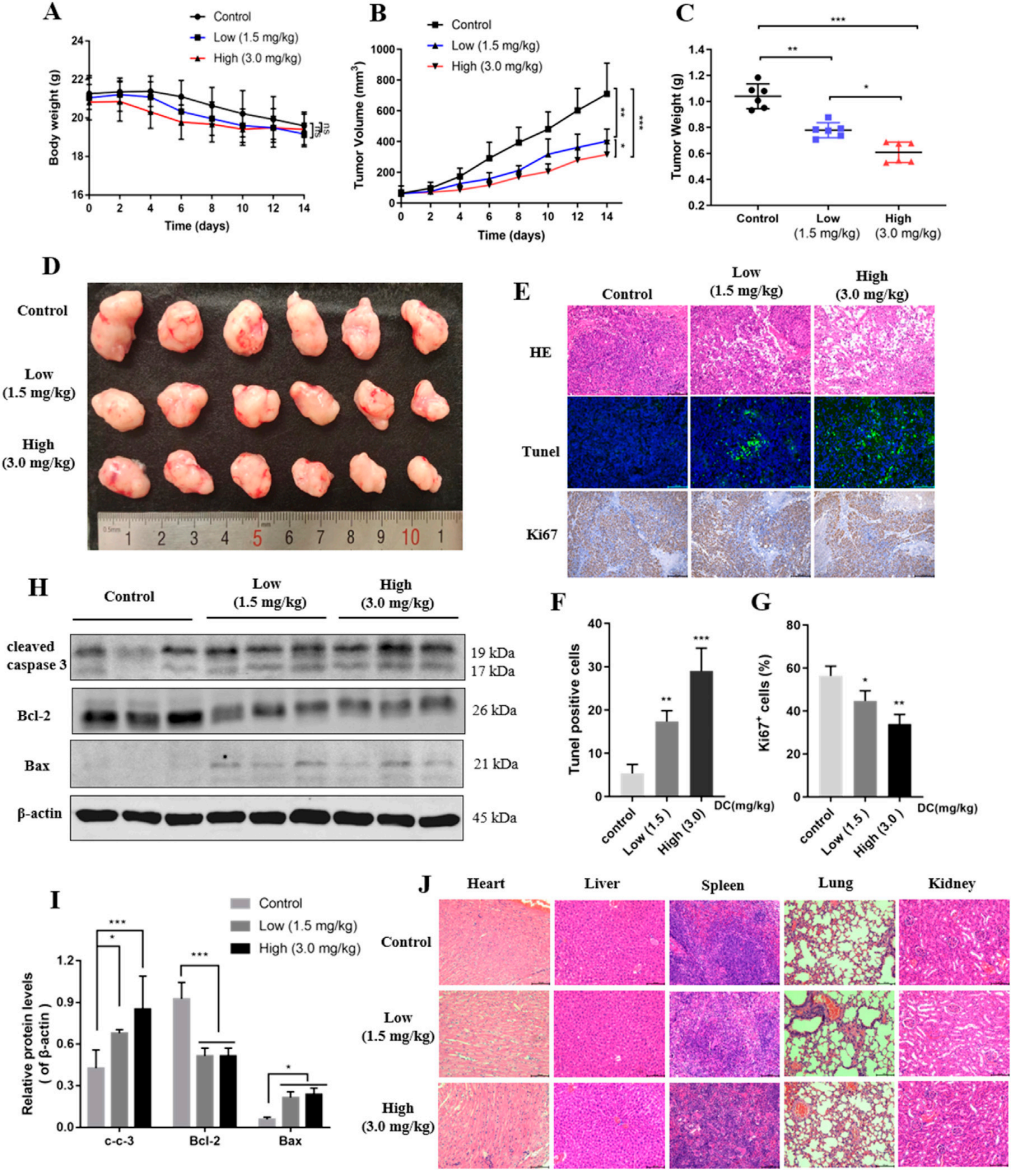
DC suppressed tumor growth in HT-29 cell xenograft mice. HT-29 cells were subcutaneously injected into the right flanks of nude mice, and saline or DC was administrated intraperitoneally daily for 14 days. **(A)** Time course of body weight (n = 6). **(B)** Time course of tumor growth progression (n = 6). **(C)** Tumor weight measured at the end of the experiment (n = 6). **(D)** Images of the excised tumors at the end of the experiment. **(E)** Tumor sections were subjected to HE, tunel and immunohistochemistry staining for Ki67. **(F,G)** The cell number of tunel and ki67 were quantitatively analyzed. **(H)** The relative protein expression levels of cleaved-caspase 3, Bcl-2, Bax compared to β-actin in the tumor tissues. **(I)** Detection of apoptosis related proteins by Western blot analysis. **(J)** Hearts, livers, spleens, lungs, and kidneys were stained with H&E. Data are expressed as the mean ± S.D. of three independent experiments. Significance was determined by the one-way ANOVA (***P < 0.001, **P < 0.01, *P < 0.05 vs. control).

The anti-tumor efficacy of DC was further examined using a HT-29 cell xenograft mouse model. When the tumor volume reached 100 mm^3^, the 0.9% sodium chloride or DC at doses of 1.5 mg/kg and 3.0 mg/kg was administered by intraperitoneal injection daily for 14 days. Mice were weighed, and tumor volumes were measured at the beginning of each treatment. The results revealed that administration of different doses of DC did not change the body weight of mice compared to the control group (Figure 7A). The tumor volume and weight were approximately 709.6 mm^3^ and 1.04 g in the control group, while the tumor volume and weight significantly decreased to 402.4 mm^3^, 315.6 mm^3^ and 0.78 g, 0.61 g following 1.5 mg/kg, 3.0 mg/kg DC treatment after 14 days (Figures 7B–D), suggesting that DC could suppress colon cancer growth *in vivo*.

HE, Tunel, and ki67 analyses were performed to observe the morphological changes of tumors. HE staining revealed large areas of necrosis in the tumor tissues of the DC-treated group, while no necrosis or only mild necrosis was observed in the control group. Tunel and ki67 experiments indicated that Tunel-positive cells (green fluorescence) were increased, and ki67-positive cells indicate proliferation was reduced after the mice were treated with 1.5 and 3.0 mg/kg DC in the tumor tissues (Figures 7E–G), respectively. The expression levels of apoptotic proteins were detected by Western blot. Consistent with the *in vitro* results, the protein expression levels of cleaved caspase 3 and Bax were significantly elevated and the Bcl-2 was reduced in the tumors after the indicated DC treatment (Figures 7H,I). These results showed that the number of apoptotic cells dramatically increased in the DC-treated group but not in the control group. In addition, no significant changes were observed in the histological morphology of the heart, liver, spleen, lung, and kidney of DC-treated mice (Figure 7J), indicating that DC was not obviously toxic to normal tissues *in vivo*.

## 4 Discussion

Over the past few years, natural products have been widely concerned as cancer treatment agents. It is believed that natural products are from a wide variety of sources and have high bioactivity and low toxicity. Accumulated research has proven that natural compounds could reduce the incidence of tumors, metastases, and invasions by inducing apoptosis and inhibiting angiogenesis. DC is a sesquiterpenoid active ingredient from the *Asteraceae* plants that has been proven to have a wide spectrum of biological effects. For example, Qin et al. (2022) have found that DC had a potent inhibition on *Staphylococcus* species by targeting the bacteria′s transmembrane channel proteins, as well as a potential therapeutic effect in the MRSA-infected mice groups *in vivo*. Additionally, DC exerts an anti-proliferative effect against HL-60 leukemia cells by activating the mitochondrial pathway and disrupting the cell cycle progression (Molnár et al., 2016). In this study, our results first demonstrated that DC suppressed cell proliferation by reducing cell viability and inhibiting colony formation in HT-29 and HCT-116 cells, indicating that DC could have potential antitumor effects.

Cellular apoptosis is a key part of the innate tumor suppression mechanism. Anticancer drugs induce tumor cell apoptosis by inactivating cells during oncogenesis. Studies have shown that the reduction of the Bcl2/Bax ratio, the loss of mitochondrial membrane potential (MMP), the overproduction of reactive oxygen species (ROS), and the activation of related caspase proteins contribute to apoptosis. For example, corylin (Yang et al., 2021), an isoflavone isolated from *Cullen corylifolium* (L.) Medik, was found to be able to inhibit colorectal cancer cell proliferation and induce apoptosis by decreasing p-STAT3/STAT3 protein levels. Inoscavin A (Qiu et al., 2022), a pyrone compound isolated from the *Sanghuangporus vaninii*, exerted antitumor effects by inhibiting the activation of the Hedgehog pathway to induce apoptosis of HT-29 cells. Moreover, punicalagin and granatin B were found to be potent antioxidants in pomegranate peels, and the mechanistic studies revealed that they could induce ROS-mediated apoptosis in HT-29 cells (Chen et al., 2022). Similar to these natural products, DC induced a significant apoptosis rate in HT-29 and HCT-116 cells in a dose-dependent manner. The Bcl-2 family members are closely associated with the mitochondria-dependent apoptotic pathways, such as the apoptotic protein Bax and the anti-apoptosis Bcl-2 protein, which are involved in suppressing caspase activation. Our results suggested that DC could induce HT-29 and HCT-116 cell apoptosis through upregulation of Bax and downregulation of Bcl-2 expression and activation of the caspase-3 pathway in HT-29 and HCT-116 cells, suggesting that apoptosis induction could play an important role for DC-mediated anti-proliferation of colon tumor cells.

Imbalance of the cell cycle can lead to tumor cell proliferation. CDKs and cyclins are the core factors of endogenous regulation and control of the cell cycle (Naeem et al., 2022). Different CDK/cyclin complexes control the different phases of the cell cycle. The CDK4/cyclin D, CDK6/cyclin D, and CDK2/cyclin E complexes regulate the G1-S phase transition, and CDK2/cyclin A and CDK1/cyclin A control the S-G2 phase progression. CDK1/cyclin B is a critical mitotic initiator. The inhibition of CDK2/cyclin A and CDK1/cyclin A complexes indicates a potential cell cycle arrest in the S phase. Some natural products have been shown to inhibit cancer cell growth by affecting mitosis and cell cycle transition, making them an alternative to chemotherapy. Sulforaphane (Wang et al., 2021b), extracted from broccoli sprouts, could significantly inhibit cell proliferation by arresting the cell cycle at the S phase and increasing the expression levels of p53 and p21, and decreasing the level of CDK2, which directly regulates the S phase transition in gastric cancer cells. 6,7,4′-THIF (Lee et al., 2011), a metabolite of daidzein, was shown to induce cell cycle arrest at the S and G2/M phases in HCT-116 cells by suppressing the expression of CDK2. In addition, S and G2/M phase arrest was found in MDA-MB-231 cells after treatment with 7,8-dihydroxy-3-arylcoumarin, which has been shown to have the highest cytotoxic activity and caused significant cell cycle arrest in the S phase and moderate arrest in the G2/M phase by incerasing the expression of cyclins A/B1, p21 and CDKs 4/6, and reducing the expression of cyclin E2 and CDK2 regulatory proteins (Musa et al., 2018). Our results indicated that DC treatment mainly increased the proportion of colon cells in S phase and decreased in G0/G1 phase, as well as induced a dose-dependent decrease of CDK2 and cyclin A_2_ expression, suggesting that the DC suppressed HCT-116 and HT-29 cell proliferation by arresting the cell cycle at the S phase in colon cells.

PPARγ is a member of the PPAR subfamily, which belongs to the nuclear receptor superfamily of ligand-inducible transcription factors and plays a key role in cellular activities. Because of its modulation of adipocyte differentiation and sensitization of adipocyte to insulin, PPARγ agonists, such as rosiglitazone and other thiazolidinediones, have been employed for the treatment of diabetes. Previous investigations have indicated that PPARγ is also a potential tumor suppressor in a variety of tissues. For example, Yang et al. (2019) have demonstrated that rosiglitazone could reduce E2F2 expression to suppress the proliferation of nasopharyngitis cell lines. Rosiglitazone and pioglitazone (Lv et al., 2019) also markedly induced cell cycle G2 arrest and apoptosis to inhibit cell proliferation in bladder cancer *in vitro* and *in vivo*. Natural products, as a major source of PPARγ agonists, play an important role in inhibiting tumor cell proliferation and cancer development. 6‐Shogaol (Tan et al., 2013), a major bioactive ingredient in the rhizomes of ginger, could induce PPARγ transcriptional activity to suppress NFκB activation and increase apoptosis in breast and colon cancer cells. Moreover, 5β,19-epoxy-19-methoxycucurbita-6,23-dien-3β,25-diol (Weng et al., 2017), a triterpenoid isolated from *M. charantia,* could inhibit cell proliferation and induce G1 cell cycle arrest in MCF-7 cells through PPARγ activation. The previous report showed that HT-29 cells expressed relatively high levels of PPARγ protein among cancer cells. Our results indicated higher PPARγ protein expression in HT-29 cells compared to HCT-116 cells, prompting the selection of HT-29 cells for subsequent experiments. Further analysis demonstrated that DC enhanced PPARγ transcriptional activity in HT-29 cells, an effect reversible by the PPARγ antagonist GW9662.

To validate whether the mechanism of action of DC is associated with PPARγ activation, PPARγ antagonist GW9662 was employed to co-treat HT-29 cells with DC. Our results have shown that after co-treatment with DC and GW9662, the proliferation inhibition, colony formation suppression and apoptosis induction of DC were completely blocked, and the cell cycle arrest at S phase was also significantly reversed in HT-29 cells, suggesting that PPARγ could be the potential target of DC to inhibit colon cancer cell proliferation. PPARγ knockdown of HT-29 cells based on the small interfering RNAs (siRNAs) technology was employed to further confirm the above speculation. Similar to the co-treatment with GW9662, the results indicated that the anti-proliferation and apoptosis induction of DC were remarkably weakened, and the cell cycle arrest at S phase and the protein expression of CDK2 and cyclin A2 were also markedly reversed in si-PPARγ HT-29 cells. Taken together, these results implied that transcriptional inactivation of PPARγ could impair DC-mediated proliferation inhibition, apoptosis induction, and cell cycle arrest in HT-29 cells.

Consistent with the *in vitro* data, the HT-29 cell xenograft mouse model confirmed that DC not only suppressed tumor growth and development but also stimulated tumor cell apoptosis *in vivo*. Moreover, the expressions of pro-apoptotic proteins Bax and cleaved caspase 3 were elevated, and the anti-apoptotic protein Bcl-2 was decreased by DC in tumor tissue. Additionally, no apparent organ damage was observed in mice when DC was administered at a dose of 3.0 mg/kg in the present study.

In this experiment, we studied the anti-tumor effect of DC through inducing the PPARγ transcription activation of colon cancer cells. However, considering that a lot of natural products, such as tanshinone, curcumin, resveratrol, and artemisinin, have been shown to have multiple anti-tumor mechanisms based on different targets and pathways, the antitumor efficacy of DC in other signaling pathway or target should warrant further investigation.

## 5 Conclusion

In conclusion, DC showed anti-tumor effects by inhibiting colon cancer cell proliferation and colony formation, inducing apoptosis, and arresting cell cycle at S phase *in vitro*, as well as suppressing tumor growth in HT-29 cell xenograft mice *in vivo*. Further mechanism study suggested that activation of PPARγ transcription was involved in DC-induced proliferation inhibition, apoptosis induction and cell cycle arrest of HT-29 cells. It was also demonstrated that DC treatment at the dose of 3.0 mg/kg could significantly suppress tumor growth and show no apparent toxicity in the xenograft nude mouse model *in vivo.* In addition, our study also supports DC as a unique PPARγ agonist for antitumor lead compounds.

## Data Availability

The datasets presented in this study can be found in online repositories. The names of the repository/repositories and accession number(s) can be found in the article/Supplementary Material.

## Abbreviations

ATCC, American Type Culture Collection; Bax, Bcl-2-associated X protein; Bcl-2, B-cell lymphoma 2; CCK, cell counting kit-8; CDKs, cyclin-dependent kinase; DAPI, 4,6-diamidino-2-phenylindole dihydrochloride; DC, dehydrozaluzanin C; DMEM, Dulbecco’s Modified Eagle Medium; DMSO, dimethyl sulfoxide; HE, hematoxylin-eosin staining; IC_50_, the half maximal inhibitory concentrations; PBS, phosphate-buffered saline; PI, propidium iodide; PPARγ, peroxisome proliferator-activated receptor γ; PVDF, polyvinylidene fluoride; Rog, rosiglitazone; SDS-PAGE, sodium dodecyl sulfate-polyacrylamide gel electrophoresis; si-RNA, small interfering RNA; TZDs, thiazolidinediones.
