# Supplementary material for: Dehydrozaluzanin C inhibits colon cancer cell proliferation, apoptosis and cycle arrest through peroxisome proliferator-activated receptor γ (PPARγ) activation
Source: Front Pharmacol. 2025 Sep 11;16:1623153. doi: 10.3389/fphar.2025.1623153 (PMC12461092; doi:10.3389/fphar.2025.1623153)
Supplement: Supplementary file 1 [file DataSheet1.pdf]

Supplementary material

Dehydrozaluzanin C inhibits colon cancer cell proliferation, apoptosis and cycle arrest through peroxisome proliferator-activated receptor γ (PPARγ) activation

Shan-Shan Li [a,b,±], Zhao-Ting Li [b,c,±], Xiao-Qing Zhu [b, c], Xu Li [b, c], Xi-Ke Xu [b], Xian-Peng Zu [b,*], Xian Li [c, *], Yun-Heng Shen [b,*]

[a] Center of Clinical Pharmacy, First Affiliated Hospital of Kunming Medical University, Kunming 650032, Yunnan, China

[b] Department of Phytochemistry, School of Pharmacy, Naval Medical University, Shanghai 200433, China

[c] School of Pharmaceutical Sciences and Yunnan Key Laboratory of Pharmacology for Natural Products, Kunming Medical University, Kunming 650500, Yunnan, China

* Correspondence

Department of Phytochemistry, School of Pharmacy, Naval Medical University, 325 Guohe Road, Shanghai 200433, P. R. China. E-mail: zuxianpeng@163.com (X.-P. Zu), yhshen@smmu.edu.cn (Y.-H. Shen)

School of Pharmaceutical Sciences and Yunnan Key Laboratory of Pharmacology for Natural Products, Kunming Medical University, Kunming 650500, Yunnan, China. E-mail: xianlikm@163.com (X. Li)

± These authors contributed equally to this work

Contents:

Supplementary Figure 1: The cytotoxicity effect in MCF-7, HT-29 and DU145 three human tumor cells……………………………………………………………………3

Supplementary Figure 2: Example of original western blot for three repeats- Fig.2-3 DC induced cell apoptosis, cycle arrest and related-protein expression in colon cancer cells. ……………………………………………………………………………… 4

Supplementary Figure 3. Example of original western blot for three repeats- Fig.5 The effect of PPARγ antagonist (GW9662) for DC-mediated antiproliferation, cell colony formation, apoptosis induction and cell cycle arrest in HT-29 cells. HT-29 cells were cotreated with GW9662 and DC for 24 h.………………………………………… 9

Supplementary Figure 4. Example of original western blot for three repeats- Fig.6 The effect of knockdown of PPARγ for DC-mediated antiproliferation, cell colony formation, apoptosis induction and cell cycle arrest in HT-29 cells. The si-NC or si-PPARγ HT-29 cells were treated with DC for 24 h……………………………………11

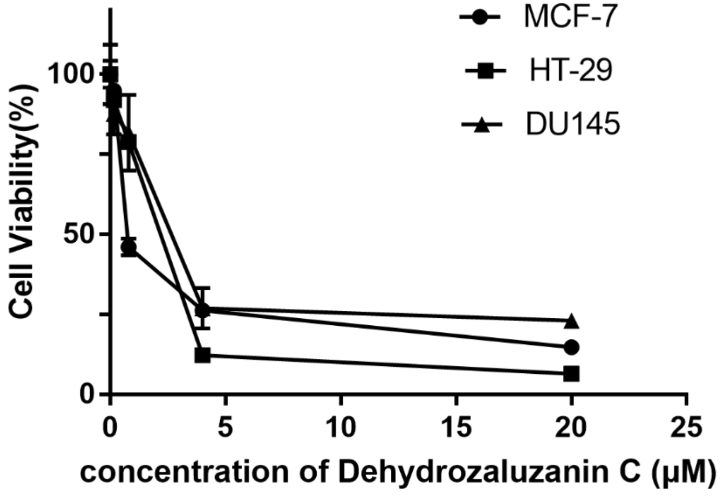

IC_{50} = 1.72±0.18 (MCF-7), 1.49±0. 05 (HT-29), 2.00±0.51 (DU145)

S Figure 1. The cytotoxicity effect in MCF-7, HT-29 and DU145 three human tumor cells.

S Figure 2. Example of original western blot for three repeats- Fig.2-3 DC induced cell apoptosis, cycle arrest and related-protein expression in colon cancer cells.

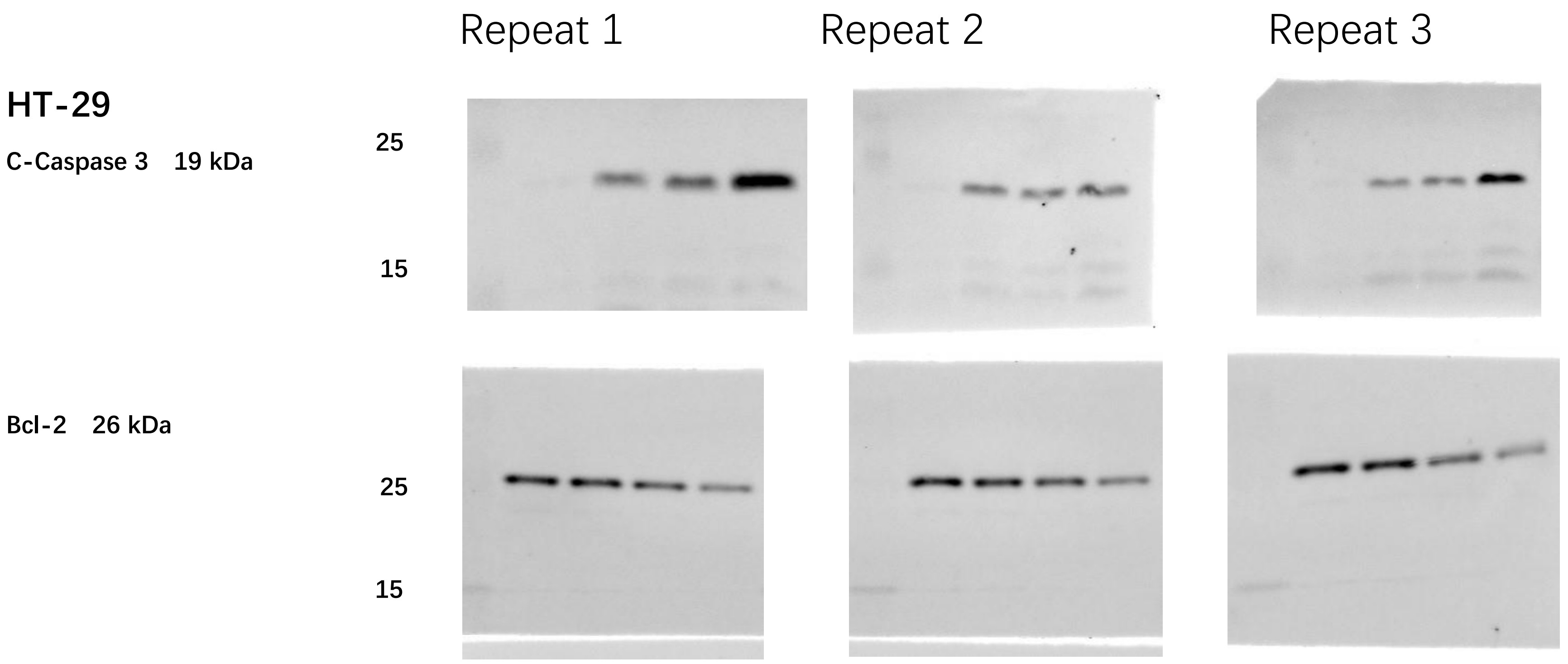

Bax 21 kDa

25
15

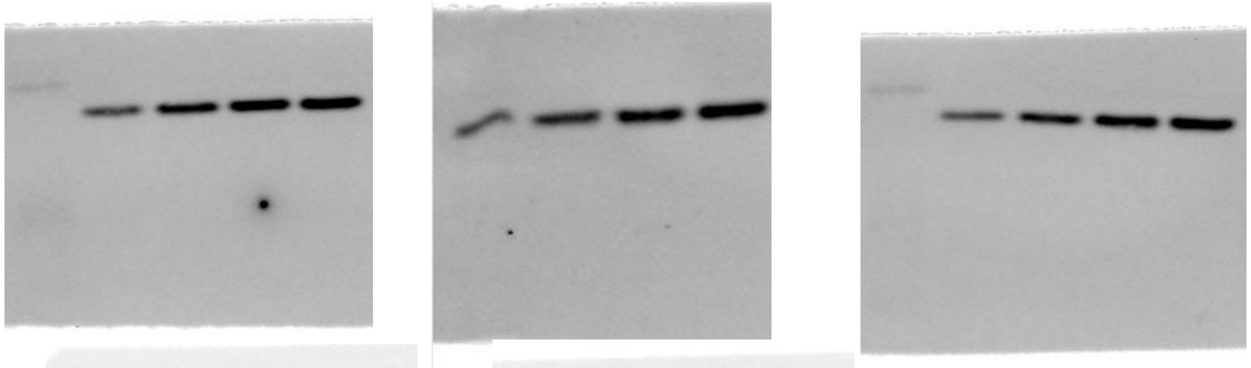

CDK2 34 kDa

40
35

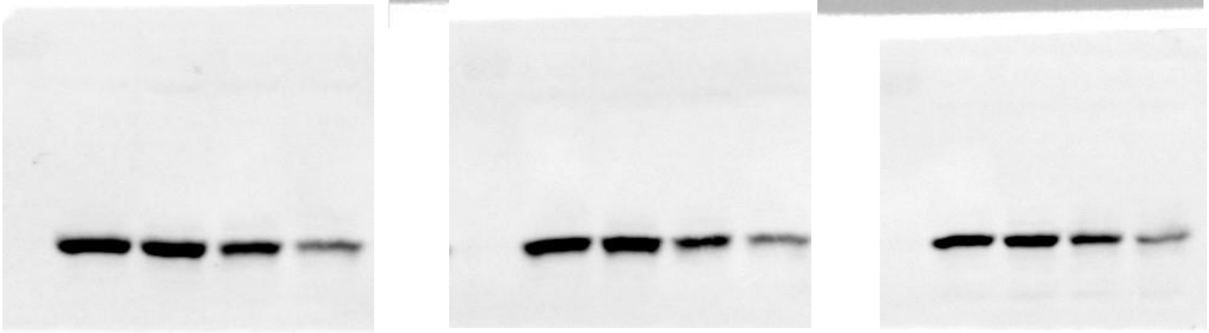

Cyclin A2 47 kDa

55
40

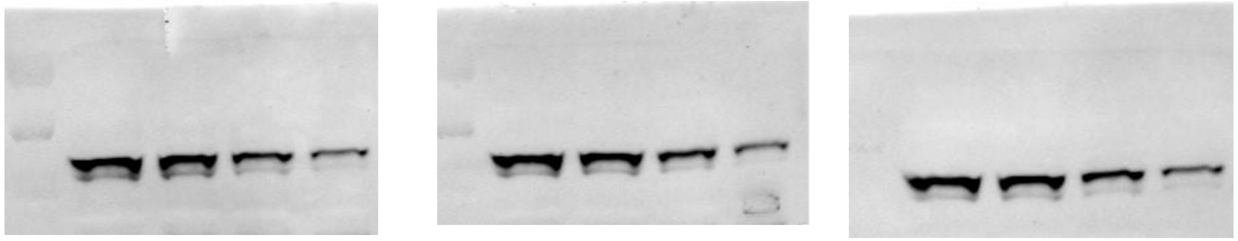

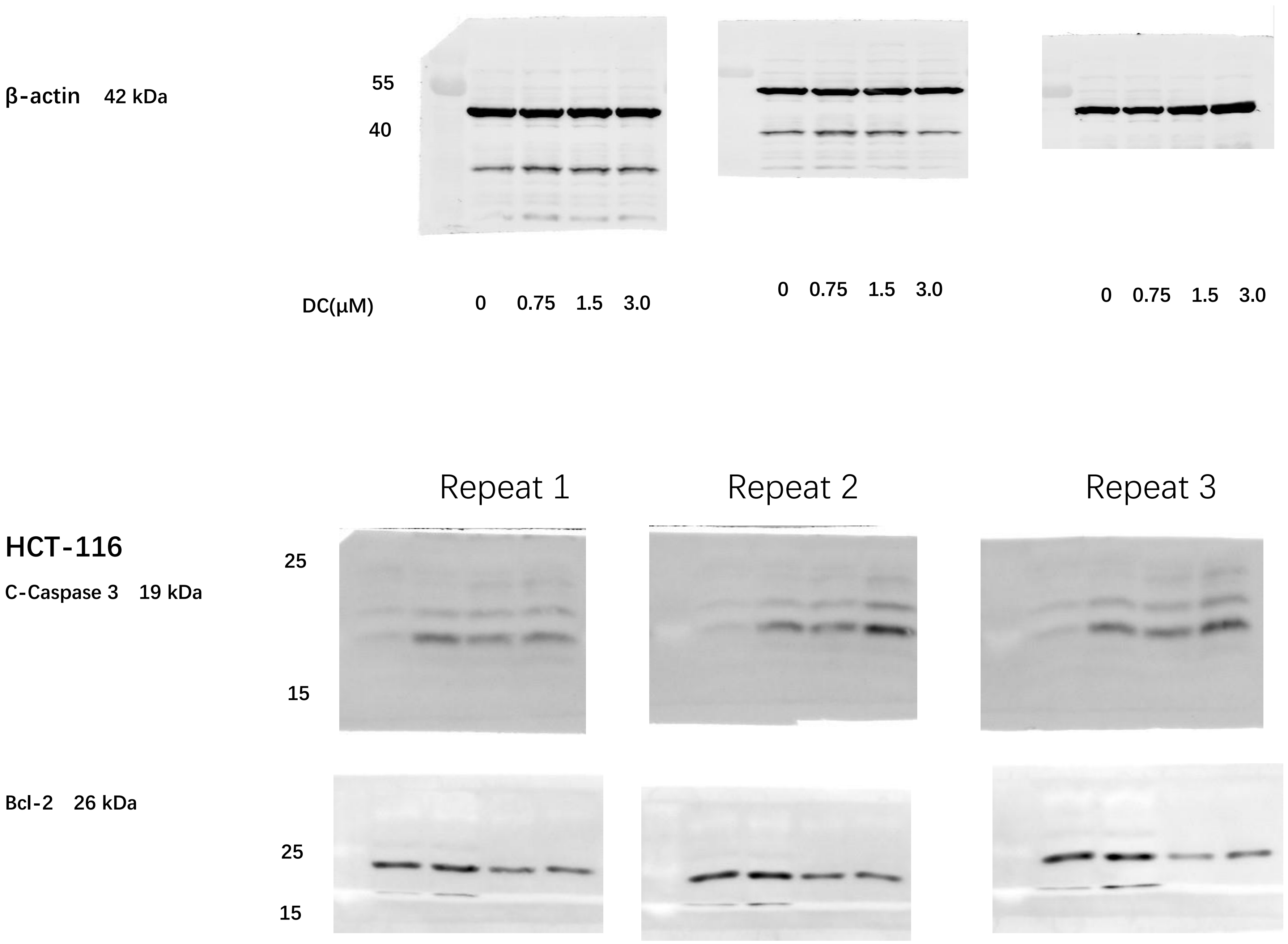
β-actin 42 kDa
55
40
DC(μM)
0 0.75 1.5 3.0
0 0.75 1.5 3.0
0 0.75 1.5 3.0
Repeat 1
Repeat 2
Repeat 3
HCT-116
C-Caspase 3 19 kDa
25
15
Bcl-2 26 kDa
25
15

Bax 21 kDa

25

15

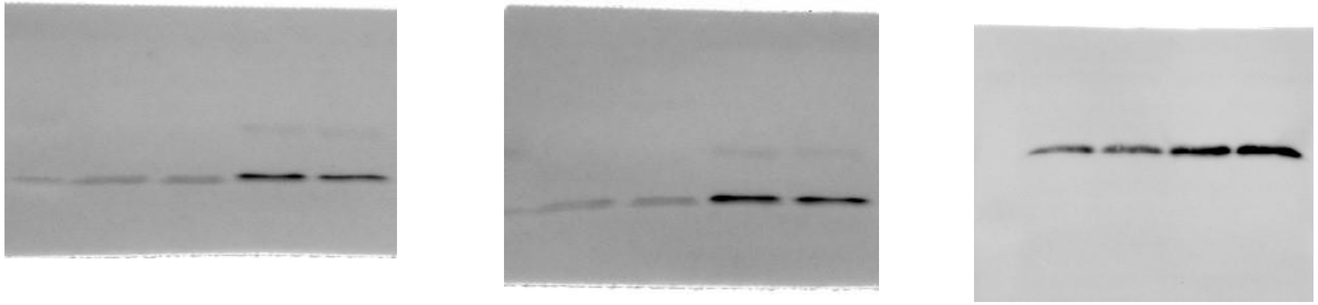

CDK2 34 kDa

40

35

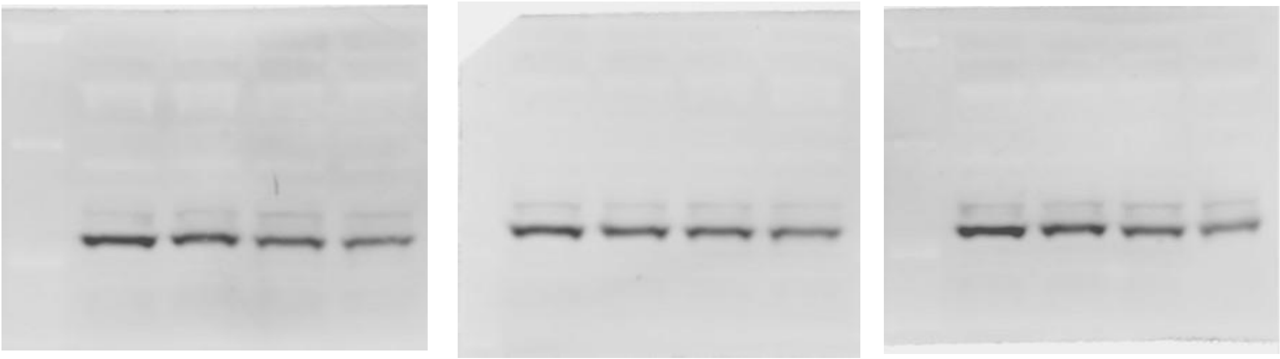

Cyclin A2 47 kDa

55

40

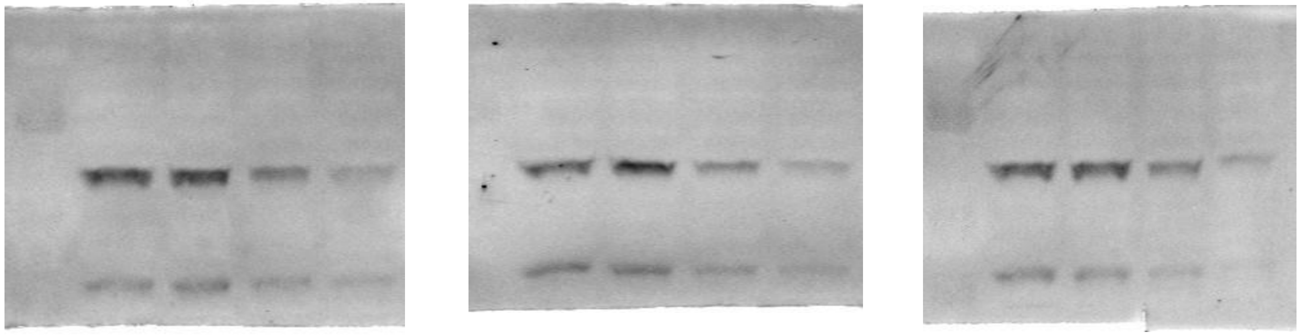

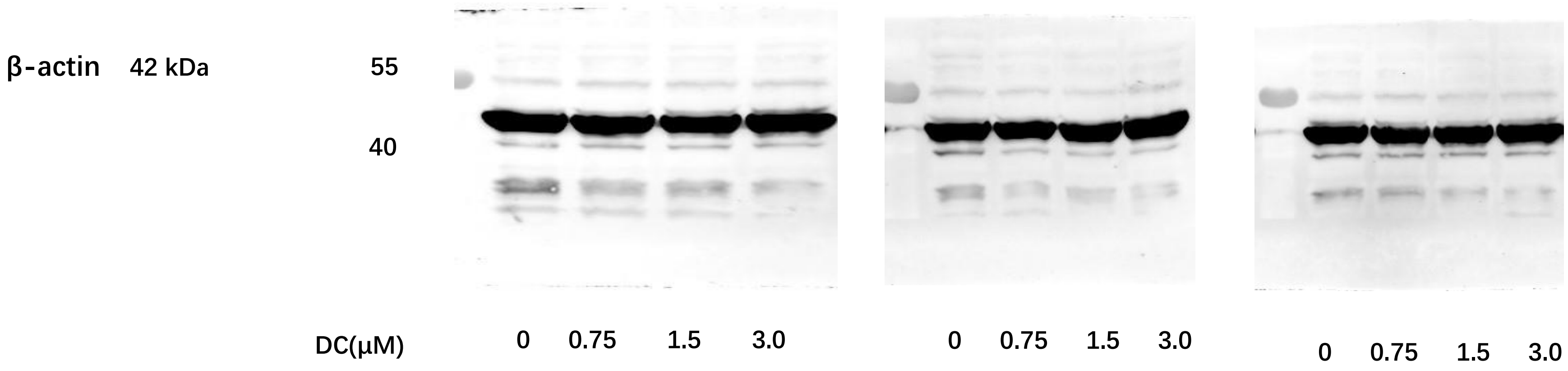
β-actin
42 kDa
55
40
DC(μM)
0
0.75
1.5
3.0
0
0.75
1.5
3.0
0
0.75
1.5
3.0

S Figure 3. Example of original western blot for three repeats- Fig.5 The effect of PPARγ antagonist (GW9662) for DC-mediated antiproliferation, cell colony formation, apoptosis induction and cell cycle arrest in HT-29 cells. HT-29 cells were cotreated with GW9662 and DC for 24 h.

HT-29 GW9662

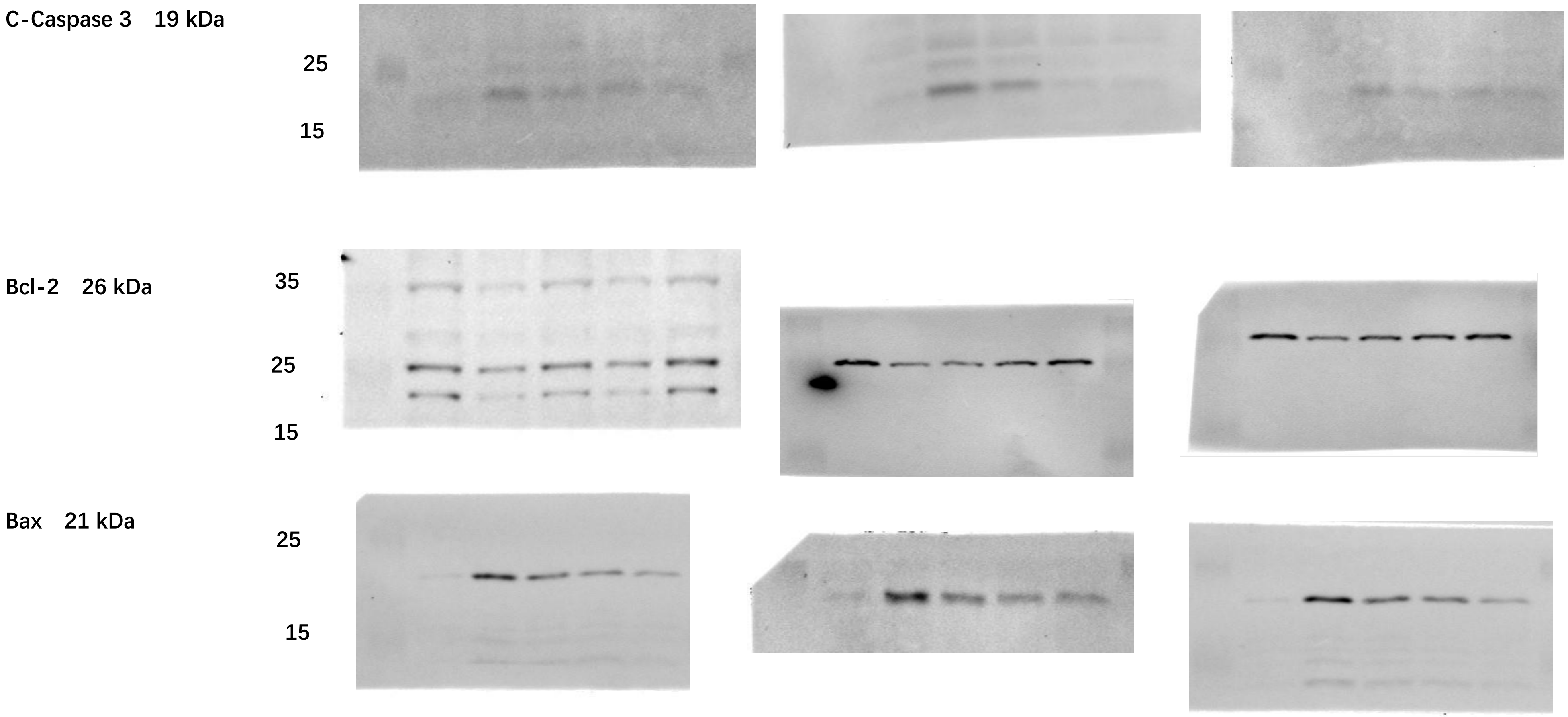

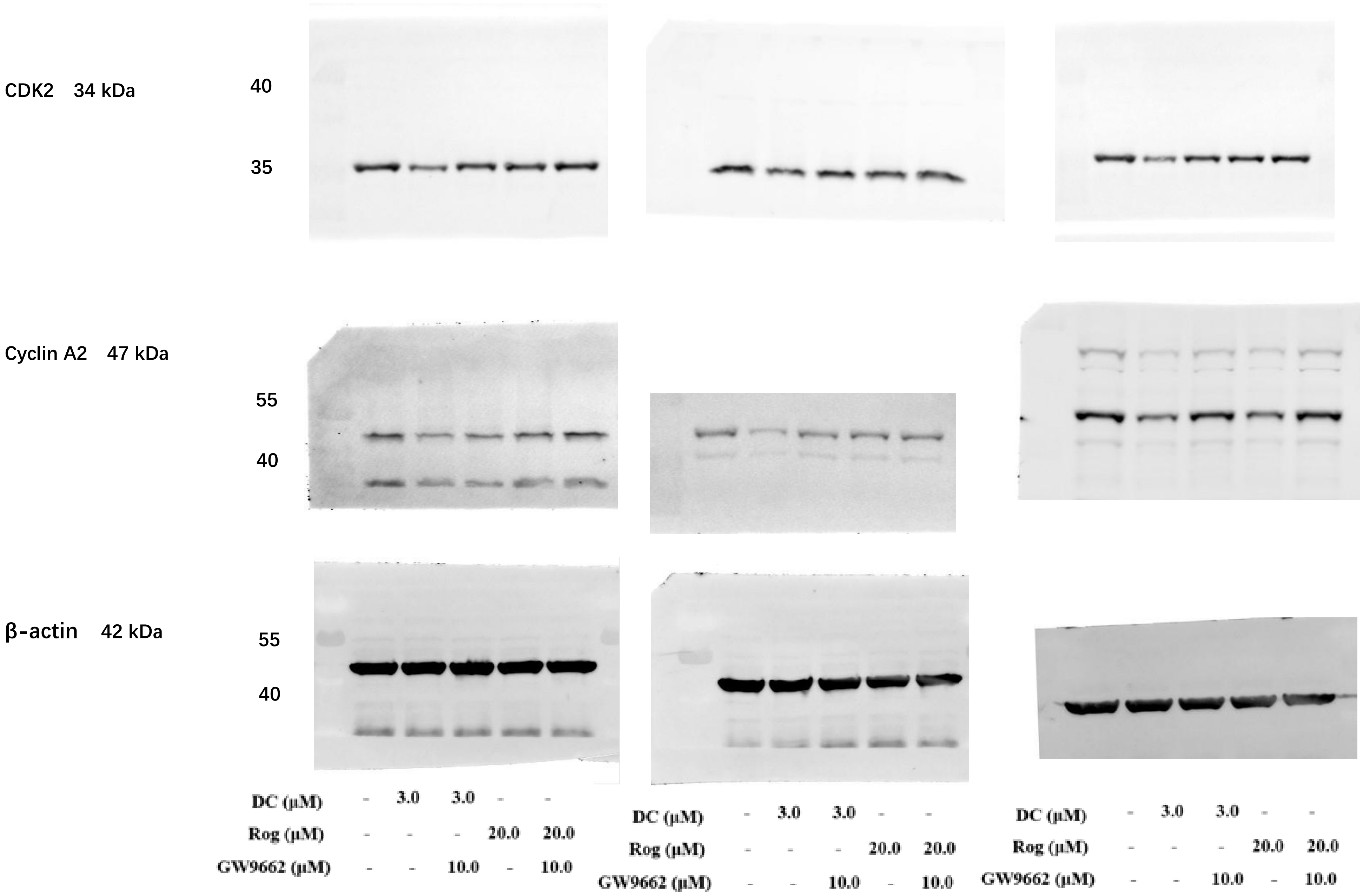
CDK2 34 kDa
40
35
Cyclin A2 47 kDa
55
40
β-actin 42 kDa
55
40
DC (μM) - 3.0 3.0 - -
Rog (μM) - - - 20.0 20.0
GW9662 (μM) - - 10.0 - 10.0

S Figure 4. Example of original western blot for three repeats- Fig.6 The effect of knockdown of PPARγ for DC-mediated antiproliferation, cell colony formation, apoptosis induction and cell cycle arrest in HT-29 cells. The si-NC or si-PPARγ HT-29 cells were treated with DC for 24 h.

HT-29 si-PPARγ

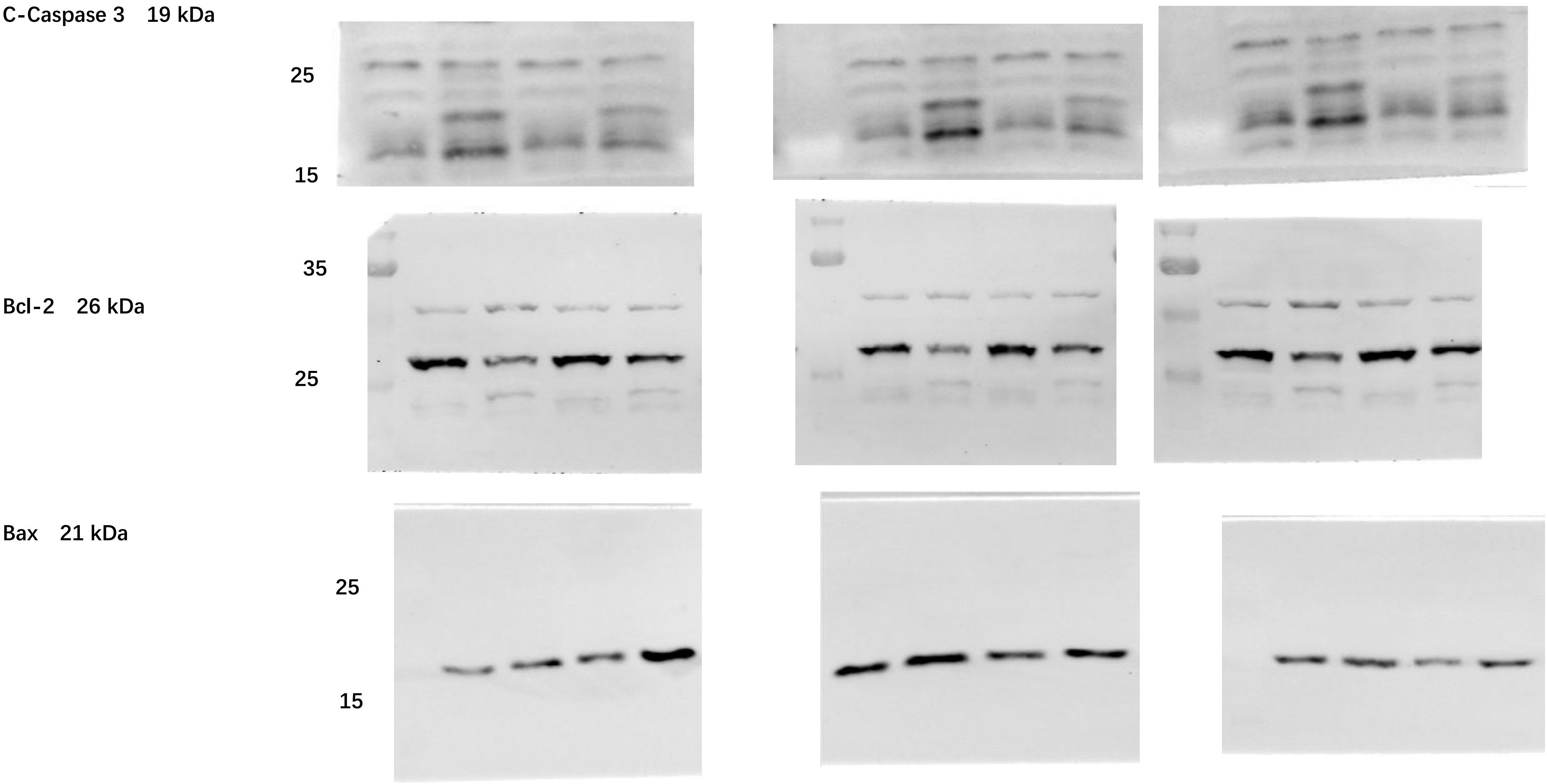

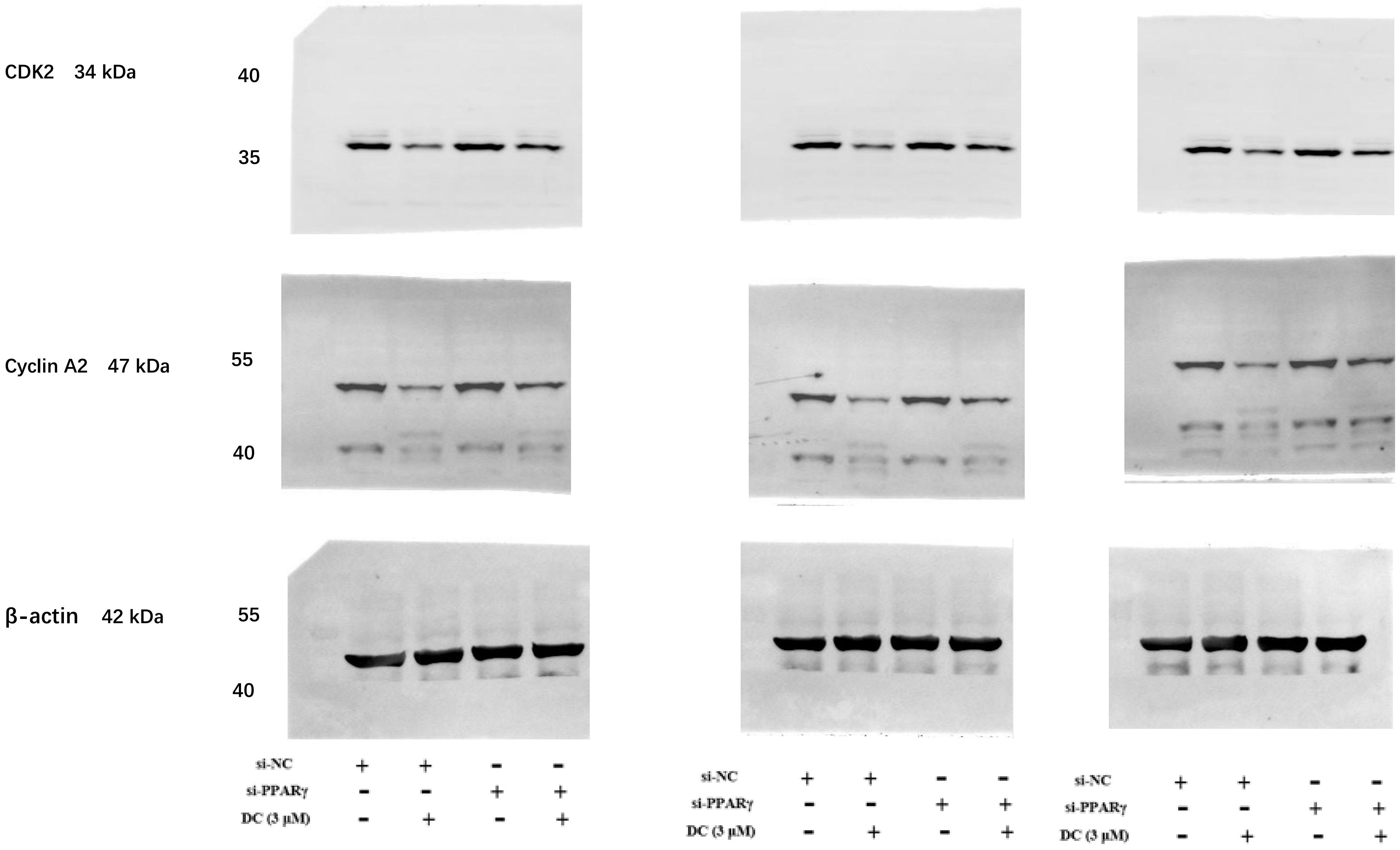
CDK2 34 kDa
40
35
Cyclin A2 47 kDa
55
40
β-actin 42 kDa
55
40
si-NC + + - -
si-PPARγ - - + +
DC (3 μM) - + - +
si-NC + + - -
si-PPARγ - - + +
DC (3 μM) - + - +
si-NC + + - -
si-PPARγ - - + +
DC (3 μM) - + - +
